# Stress-Dependent NF-κB Signaling in Acute Kidney Injury: Linking Inflammation, Autophagy, and Apoptosis

**DOI:** 10.3390/ijms27114960

**Published:** 2026-05-29

**Authors:** Dev Kumar

**Affiliations:** Division of Nephrology, Department of Internal Medicine, Carver College of Medicine, University of Iowa, Iowa City, IA 52242, USA; devkumar@uiowa.edu

**Keywords:** NF-κB, acute kidney injury, inflammation, oxidative stress, autophagy, apoptosis

## Abstract

Nuclear factor-κB (NF-κB) is a critical regulator of inflammation and stress response signaling in acute kidney injury (AKI). Increasing evidence demonstrates that NF-κB signaling is directly related to oxidative stress, autophagy, mitochondrial malfunction, and apoptosis in the process of AKI. Injury-related stimuli, including ischemia–reperfusion, sepsis, nephrotoxins, reactive oxygen species (ROS) and damage-associated molecular patterns (DAMPs), activate canonical and non-canonical NF-κB pathways, resulting in renal inflammation and tubular injury. Recent investigations have shown that TLR4/NF-κB signaling, NLRP3 inflammasome activation, defective autophagy, and mitochondrial dysfunction mediate inflammatory and pro-apoptotic responses in AKI. On the other hand, autophagy-associated proteins such as microtubule-associated protein 1 light chain 3 beta (LC3B) and Beclin-1 may play renoprotective roles through the regulation of NF-κB signaling. This review tries to cover the knowledge regarding NF-κB signaling in AKI and to emphasize the possible function of NF-κB signaling in the control of inflammation, autophagy, and apoptosis. It also seeks to provide some insight into future research directions that may guide the development of more effective therapies for AKI.

## 1. Introduction

Acute kidney injury (AKI) is defined as a critical medical condition characterized by sudden loss of renal function. It increases serum levels of nitrogenous and non-nitrogenous products, resulting from defects in the kidney excretion of waste products [1,2]. The multifaceted etiology of AKI, due to exposure to nephrotoxins, renal ischemia, congestive heart failure, and septic shock, results in an immense impact on global health that affects approximately 13.3 million people worldwide, with a mortality of approx. 2 million per year, i.e., 23.9% and 13.8% in adults and children, respectively [3,4,5]. Recent studies have demonstrated that, besides inflammation, other processes, such as oxidative stress, apoptosis, and autophagy, also play important roles in AKI. Nuclear factor-κB (NF-κB) functions as a central molecule orchestrating these critical cellular processes whose altered function led to renal cell damage in AKI [6,7,8]. Since its discovery as a nuclear factor binding to the kappa enhancer of κ light chain of immunoglobulin in 1986 by David Baltimore and Ranjan Sen, NF-κB has been believed to be a primary regulator of inflammation, controlling various other cellular responses, such as cell proliferation, autophagy, and apoptosis [9,10,11,12,13,14]. Likewise, its role in the pathogenesis of inflammatory diseases in the kidney has been validated in animal models of renal inflammatory disease and human kidney disease. Also, NF-κB’s activation is vital for the initial inflammatory response of innate immune cells towards injury. A wide range of kidney injury-related stimuli activate NF-κB; these include growth factors, cytokines such as tumor necrosis factor-alpha (TNF-α), interleukin-1 beta (IL-1β), and interleukin-6 (IL-6), damage-associated molecules, genotoxic stress, immune mediators, phorbol esters, chemotherapeutic drugs, hypoxia, ionizing radiation, mechanical stress, and bacterial products such as lipopolysaccharides (LPS) [15,16]. Indeed, many publications have reported the implication of NF-κB deregulation in various pathologic conditions [17,18,19]. However, the extent of inflammatory response and the mechanism of NF-κB during AKI and the extent of injury that leads to its progression to chronic kidney disease (CKD) remain incompletely understood. As NF-κB is involved in various cellular processes, its global inhibition may also lead to severe side effects. Hence, this review aims to integrate recent knowledge of NF-κB regulation and its role in AKI pathophysiology, and to suggest new potential therapeutic targets for AKI treatment.

## 2. NF-κB Structure and Signaling Pathways

The Rel/NF-κB family, initially identified as nuclear transcription factors bound to the enhancer of the κ-immunoglobulin light chain gene in mature B cells, comprises structurally related proteins across various organisms. Comprising RelA (p65), RelB, c-Rel, NF-κB1 (p50/p105), and NF-κB2 (p52/p100) encoded by *RELA*, *RELB*, *REL*, *NFKB1*, and *NFKB2*, respectively. NF-κB signaling regulates cellular processes such as inflammation, cell survival, cell migration, apoptosis, and stress responses [20,21,22]. Structurally, the NF-κB family of proteins shares a conserved Rel homology domain (RHD) responsible for dimerization, DNA binding, and nuclear localization. Based on their transactivation potential, they are divided into two groups: RelA, RelB, and c-Rel, which contain the C-terminal transactivation domain (TAD) and function as transcriptional transactivators, whereas the other group, p50 and p52, lacking the TAD, primarily regulate gene expression via dimerization with other subunits [23].

Activation of NF-κB proteins is controlled by signaling cascades involving IκB kinase (IKK) complex, having two catalytic subunits (IKKα, IKKβ) and a regulatory subunit (IKKγ/NEMO) [24,25,26,27,28,29,30]. Upon stimulation, a myriad of intracellular and extracellular factors such as microbial products, pro-inflammatory cytokines, or reactive oxygen species activate the IKK complex, which phosphorylates IκB proteins, leading to their ubiquitination and proteasomal degradation, resulting in the release of NF-κB proteins and their translocation into the nucleus, where they regulate various genes involved in inflammation, immune response and cell cycle regulatory genes [31,32,33].

**Figure 1:**
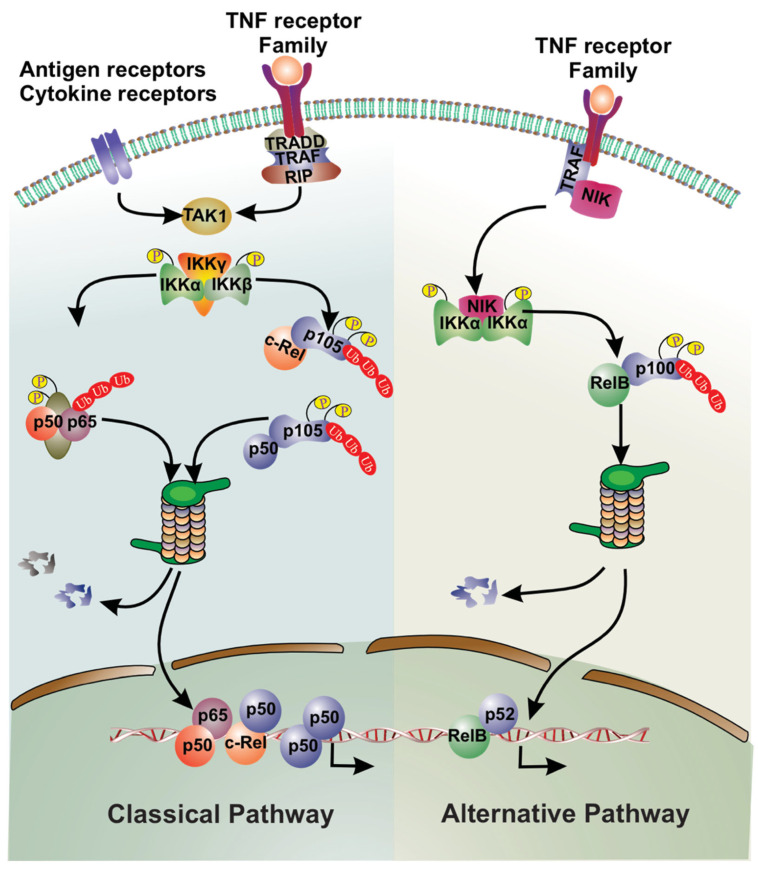
Canonical and non-canonical NF-κB signaling pathways. NF-κB activation is initiated by ligand binding to cell-surface receptors, leading to the recruitment of adaptor proteins such as TRADD, TRAF, and RIP. In the canonical pathway, activation of the IKK complex—primarily the IKKβ subunit—results in phosphorylation and proteasomal degradation of IκB, allowing the p65/p50 heterodimer to translocate to the nucleus and regulate target gene expression. In the non-canonical pathway, activation of NF-κB-inducing kinase (NIK) leads to phosphorylation of IKKα homodimers, which in turn phosphorylate p100, promoting its processing into p52 and formation of the RelB/p52 complex. This complex subsequently translocates to the nucleus to regulate gene transcription. Ub, ubiquitination; P, phosphorylation.

NF-κB signaling occurs via two distinct pathways: classical (canonical) and alternative (non-canonical) pathways (Figure 1). The classical pathway is initiated by a broad range of stimuli, including pathogen-activated signals, interleukins, and tumor necrosis-α (TNF-α), and involves the activation of p65/p50 homodimer following degradation of IκB [34]. On the other hand, the alternative pathway is activated by a limited number of stimuli and relies on Nuclear factor kappa-B-inducing kinase (NIK), which facilitates proteasome-mediated processing of p100 to p52, thereby releasing the active RelB-p52 complex. This active complex enters the nucleus, driving transcriptional regulation of target genes distinct from the IκBα-regulated canonical NF-κB pathway. This pathway regulates genes involved in immune cell development and lymphoid organogenesis [35,36]. Collectively, these pathways enable NF-κB to serve as a critical signaling hub that integrates diverse environmental signals and coordinates transcriptional programs vital to cellular homeostasis and disease development.

## 3. NF-κB Signaling in AKI

Besides its role in inflammation, NF-κB is reported to mediate other important cellular functions in the setting of AKI, including cell cycle, apoptosis, autophagy, and senescence [37,38]. Several stimuli associated with kidney injury, ranging from growth factors and cytokines (such as TNF-α, IL-1, and IL-6) to damage-associated molecules, genotoxic stress, and bacterial products like LPS, can activate the NF-κB signaling in AKI [39,40,41]. Depending on the type of stimuli, NF-κB activation in AKI occurs via the canonical (or classical) and non-canonical (or alternative) pathways. Accumulating evidence suggests that both pathways play roles in the pathophysiology of AKI. For instance, research on Shiga-toxin-2-induced acute kidney injury in mice implicates the alternative NF-κB signaling pathway [42]. The TNF family cytokines TNF and TWEAK have been shown to induce different pathways of NF-κB activation in renal tubulointerstitial inflammation [43,44]. In this condition, the transient canonical NF-κB pathway is mainly activated by TNF, which is generally characterized by the nuclear translocation and binding of RelA to the DNA (Figure 1). On the other hand, TWEAK activates both canonical and non-canonical pathways. In the early stages of injury, it activates the RelA-dependent canonical pathway, and later, it activates the p52/RelB-dependent non-canonical pathway and facilitates its nuclear translocation and DNA binding [45].

### 3.1. Inflammation

The pathophysiology of kidney diseases involves immune and inflammatory factors [46,47]. AKI pathogenesis is significantly influenced by inflammation. Kidney injury is significantly aggravated by AKI-induced inflammation, and reducing inflammation has been shown to reduce kidney injury and speed up recovery [35,39]. It is thought that NF-κB plays a key role in mediating inflammation and is activated in conjunction with kidney injury induction caused by ischemia–reperfusion [48]. In animal models, NF-κB inhibitors have been shown to reduce renal inflammation and injury [49,50]. Generally, NF-κB signaling starts with the stimulation by various factors, cytokines, chemokines, and DAMPs. These molecules bind to their respective receptors on cells and control the activation of NF-κB, leading to the translocation of the activated form of NF-κB to the nucleus, where it regulates the transcription of genes involved in the recruitment and activation of immune cells and further exacerbates renal inflammation in AKI. A growing body of research indicates that AKI-associated renal inflammation may persist long after acute injury has subsided and renal function has recovered, potentially leading to gradual renal fibrosis [51,52,53]. The AKI inflammatory response involves activation of innate and adaptive immune cells, including neutrophils, macrophages, dendritic cells, and lymphocytes [54]. Activation of these immune cells results in the production of a range of inflammatory molecules and their respective receptors, including DAMPs, cytokines, hypoxia-inducible factors, and Toll-like receptors (TLRs) [55,56]. Table 1 summarizes the activation patterns and biological effects of NF-kB signaling in various AKI models.

### 3.2. Innate Immune Response

The innate immune cells comprising macrophages, dendritic cells, and neutrophils substantially affect the inflammation and innate immune response related to AKI. These cells produced pattern recognition receptors (PRRs), which are crucial components of the innate immune system. PRRs recognize patterns in molecular data linked to pathogens (PAMPs) and damage (DAMPs), and they use these signals to initiate downstream signaling pathways, including NF-κB activation. Transforming growth factor-β-activated kinase 1 (TAK1) is a crucial integrator of PRR signaling to NF-κB. TAK1, in conjunction with its regulatory components TAB1 and TAB2, stimulates the IκB kinase (IKK) complex. TAB2 interacts with poly-ubiquitin chains, which are necessary to activate TAK1. Upon activation, TAK1 initiates phosphorylation and activation of IKK, which subsequently phosphorylates IκBα. Once IκBα is phosphorylated, it is degraded, and NF-κB may translocate to the nucleus to initiate transcription of genes involved in the inflammatory response.

PRRs are categorized into five families: toll-like receptors (TLRs), RIG-I-like receptors, NOD-like receptors (NLRs), C-type lectin-like receptors, and cytosolic DNA sensors. These PRRs share identical downstream signaling pathways, primarily the activation of the canonical NF-κB pathway, despite their structural variations and the range of PAMPs and DAMPs they identify. This route is essential for activating pro-inflammatory cytokines, chemokines, and other inflammatory mediators at the transcriptional level. Studies have demonstrated that inhibiting TLR4/NF-κB activation can reduce acute kidney injury (AKI) [59]. In this regard, another study of the TLR-4/NF-κB pathway revealed alterations in TLR-4/NF-κB/iNOS and Nrf-2/hemeoxygenase-1 signaling pathways in rhabdomyolysis in rats [64]. A recent study showed that inhibition of the TLR4/MyD88/NF-κB signaling pathway can decrease inflammation and mitigate cell apoptosis [66]. This suggests that it has the potential to be developed as a preventive mechanism for renal ischemia-induced AKI.

### 3.3. Adaptive Immune Response

The role of NF-κB in the adaptive immune response during AKI is critical. It spans its involvement in activating immune cells to regulate cytokine production. The NF-κB-regulated adaptive immune response in AKI is activated in multiple ways, primarily through T-cell activation and differentiation. It facilitates the expression of cytokines such as IL-6 and IL-12, which are essential for activating T cells by APCs (antigen-presenting cells), including dendritic cells. The NF-κB pathway contributes to the differentiation of CD4+ T cells into T-helper cells (Th), such as Th1 and Th17, which may mediate the inflammatory response in AKI [67]. Additionally, NF-κB induces immune cells to produce IL-12 and IL-23, thereby facilitating the differentiation of CD4+ T cells into Th1 and Th17 cells, respectively. The canonical NF-κB pathway also contributes to the development of Th1 and Th17 cells within T cells [68]. Likewise, CD4+ T-helper (Th) cells are crucial in mediating inflammatory responses in AKI. Furthermore, activated CD4+ T cells generate a specific subset of immunosuppressive T cells, the inducible T regulatory (Treg) cells. Nevertheless, NF-κB also promotes the development of Treg cells, underscoring its dual role in both inflammation and immune control. Targeting NF-κB pathways can balance the roles of effector and regulatory T cells, reducing inflammation and facilitating kidney healing in cases of AKI. Further investigation is required to fully understand the role of T regulatory cells (T reg) in AKI.

Research indicates that reducing inflammation can prevent further kidney damage while encouraging a slow recovery from AKI-induced inflammation [69]. Controlling the NF-κB pathway, which is essential for AKI progression, may influence its severity. To support this, a recent study demonstrated that mitigating NF-κB signaling by calcium dobesilate ameliorates renal inflammation in a sepsis-related AKI model [70]. Another study has reported that NF-κB stimulates COX-2 synthesis in kidney cells, which is essential for AKI progression, and inhibiting COX-2 by a known inhibitor of COX-2 and NF-κB, which is Thalidomide, ameliorated the renal function in glycerol-induced AKI in rats [71]. Therefore, NF-κB’s complex role in mediating innate and adaptive immune responses to drive inflammation during AKI suggests its relevance as a therapeutic target for modulating AKI-associated inflammation and tissue damage.

### 3.4. Oxidative Stress

Oxidative stress is a key player in the pathology of AKI and plays a significant role in tubular damage, inflammation, and mitochondrial malfunction. The ischemia, sepsis, nephrotoxic injury and inflammatory activation may lead to an overproduction of reactive oxygen species (ROS), defective antioxidant system, and activation of cell death pathways, which generally lead to oxidative stress, causing direct damage to cellular proteins, lipids and DNA and activation of NF-κB signalling pathways. All these pathways are directly or indirectly linked to the NF-κB signaling. Activated NF-κB further increases the production of inflammatory cytokines, chemokines and immune-cell recruitment, resulting in a positive feedback loop between oxidative stress and inflammation. Furthermore, NF-κB is associated with increased production of enzymes that produce ROS like inducible nitric oxide synthase (iNOS) and NADPH oxidase (NOX) [72,73]. On the other hand, NF-κB also contributes to an increase in ROS by suppressing the antioxidant defense mechanisms by downregulating antioxidant enzymes, such as superoxide dismutase (SOD), catalase, and glutathione peroxidase (GPx) [74,75,76]. Thus, renal cell oxidative damage in AKI is exacerbated by the imbalance between the production of reactive oxygen species (ROS) and the antioxidant defense systems’ capacity to neutralize them. In the setting of AKI, inflammatory signals activate NF-κB signaling, which promotes the secretion of proinflammatory cytokines, including TNF-α and IL-6, and further leads to the generation of ROS in renal cells.

Oxidative stress in AKI is characterized by complex bidirectional relationships between ROS production and NF-κB signaling. In AKI, depending on cellular context, injury model and stage of injury, NF-κB can act as a main inflammatory driver, as well as a subsequent responder to ROS-mediated injury or amplifier of oxidative stress signaling. Mitochondrial dysfunction and ROS generation triggered by NADPH oxidase (NOX) are key contributors to oxidative damage and may further encourage NF-κB activation via positive feedback mechanisms [77,78]. On the other hand, antioxidant defense pathways, in particular Nrf2-mediated signaling, could compensate for ROS accumulation and attenuate overactivated inflammatory responses [79,80]. Emerging data further suggest that transitory NF-κB activation may initially enhance adaptive stress responses, but persistent activation can amplify inflammation, cause mitochondrial injury, and lead to maladaptive tubular injury [36,81]. Moreover, NF-κB activation and oxidative stress in AKI can trigger cell death pathways, such as apoptosis and necroptosis, particularly in response to oxidative stress [58,82]. Accumulation of ROS also impairs mitochondrial function, leading to the release of proapoptotic factors that initiate apoptotic cell death. Similar NF-κB-mediated oxidative and inflammatory pathways have been observed in systemic inflammatory and metabolic disorders, providing further evidence for the broader pathophysiological importance of NF-κB signaling beyond kidney injury [83]. Hence, future research targeting inflammation and oxidative stress, and altering NF-κB signaling pathways, could be a promising approach to developing therapeutic options to maintain renal function in AKI.

### 3.5. Apoptosis

In Acute Kidney Injury (AKI), NF-κB is essential for controlling apoptosis; it is a two-edged sword that mediates pathways leading to cell death and survival. During the early stages of AKI, NF-κB activation can benefit by stimulating the expression of anti-apoptotic genes [84]. These genes are essential in preserving the integrity and function of cells. Nevertheless, repeated or excessive NF-κB stimulation might shift the balance toward apoptosis, leading to kidney damage. This occurs by activating pro-apoptotic genes such as Bax, Fas, and the caspase family, which carry out programmed cell death [61,85]. A recent study has shed light on NF-κB’s intricate role in AKI. It was reported that the deliberate suppression of NF-κB using specialized inhibitors mitigates renal cell death in renal tubular epithelial cells, resulting in improved kidney function in AKI animal models [86]. Another study in mice also revealed a strong correlation between NF-κB-mediated inflammatory signaling and apoptotic pathways during AKI [87].

Recent studies have demonstrated the role of stress-dependent NF-κB signaling in regulating different stages of apoptosis during AKI. Activation of NF-κB occurs in conditions such as ischemia–reperfusion injury, nephrotoxic insult, oxidative stress, and mitochondrial dysfunction, which lead to the production of pro-inflammatory cytokines and apoptotic mediators, and subsequently exacerbates renal tubular injury [88,89,90]. This stress-induced NF-κB activation alters Bax/Bcl-2 balance, caspase-3 activation, cytochrome c release, and ROS accumulation to modulate mitochondrial apoptotic pathways [91,92]. Furthermore, an association of the NF-κB signaling pathway with TLR4 and NLRP3 inflammasome activation, causing impaired autophagy and mitochondrial metabolic dysfunction, creates an environment that leads to persistent inflammation and a pro-apoptotic environment during the development of AKI [38,40,63,73,78]. Importantly, pharmacological blockage of NF-κB-related signaling pathways has been shown to reduce tubular apoptosis, inflammatory injury, and improve renal function in experimental AKI models, suggesting that NF-κB is a promising therapeutic target for stress-associated renal injury [86,87,93,94,95].

**Figure 2:**
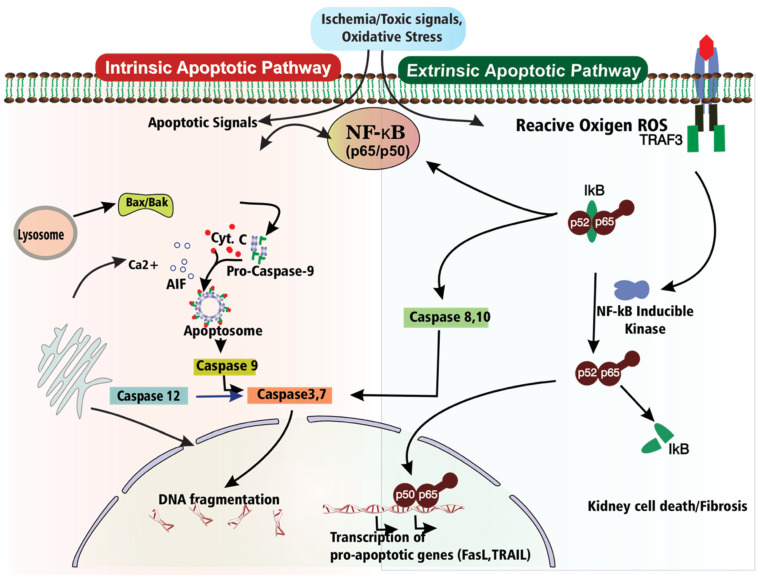
NF-κB-dependent apoptotic pathways in acute kidney injury (AKI). NF-κB contributes to apoptotic cell death through both intrinsic (mitochondrial) and extrinsic (death receptor-mediated) pathways during AKI. In the intrinsic pathway, mitochondrial dysfunction leads to the activation of pro-apoptotic proteins such as Bax/Bak, the release of cytochrome c, and the subsequent activation of caspase-9 and executioner caspases. In the extrinsic pathway, activation of death receptors and adaptor proteins promotes caspase-8 and caspase-10 activation. These pathways converge on caspase-3 and caspase-7, leading to tubular epithelial cell death and the progression of kidney injury.

In general, two pathways lead to cellular apoptosis in AKI: the intrinsic pathway involves cellular organelles such as lysosomes, the endoplasmic reticulum, and mitochondria, whereas the extrinsic pathway involves death receptor activation in response to external death signals. Both these pathways generally operate by activating some executioner proteases (Caspase-3 and Caspase-7), resulting in changes in cell morphology, i.e., cell shrinkage, membrane blebbing, and nuclear fragmentation (Figure 2). NF-κB mediated extrinsic pathway apoptosis is triggered by binding of extracellular signal proteins, viz., death ligands (Fas Ligand (FasL), TNF (Tumor Necrosis Factor), and TRAIL (Tumor Necrosis Factor-related Apoptosis-inducing Ligand) to their respective cell-surface death receptors (TNFR, Fas, and DR4/5). Upon stimulation, a cytoplasmic portion of the receptor trimerizes and recruits some adaptor protein (FADD), leading to activation of initiator caspases 8 and 10 via DED in their pro-domains to form the Death-Inducing Signal Complex (DISC). Activated initiator caspases activate the downstream executioner caspases (3, 6, and 7), which ultimately execute their effects, leading to apoptosis. Studies employing nephrotoxic insult demonstrated that TNF and Fas activation induce apoptosis in stressed renal tubular epithelial cells [96,97].

Instead of caspase-dependent apoptosis pathways, some caspase-independent apoptosis pathways do exist in nephrotoxic-induced AKI. An additional atypical pathway is also triggered upon the activation of TNFα or other related receptors, which induces apoptosis in AKI. In this regard, activation of TNFα receptors results in the assembly of intracellular adaptor proteins, such as tumor necrosis factor receptor-associated factor 3 (TRAF3). Usually, TRAF3 controls NIK degradation by engaging the E3 ubiquitin ligase complex, which flags NIK for proteasomal degradation, thereby maintaining its low basal levels. However, upon pathway activation, part of the ubiquitin ligase complex (i.e., the cellular inhibitor of apoptosis proteins (cIAPs) degrades TRAF3. The degradation of TRAF3 weakens its inhibitory effect on NIK, leading to NIK accumulation within cells [98]. The accumulated NIK phosphorylates and activates IKKα, an essential component of the IκB kinase (IKK) complex. Activated IKKα eventually phosphorylates p100, a precursor of the NF-κB subunit in the non-canonical NF-κB pathway. Phosphorylation of p100 triggers partial proteasomal processing into the active subunit p52, which associates with RelB to form an active RelB-p52 heterodimer. This active complex finally migrates to the nucleus, driving the transcription of target genes distinct from those driven by the canonical NF-κB pathway. Notably, the RelB-p52 complex can enhance pro-apoptotic signaling under certain conditions, thereby facilitating the expression of genes such as Fas ligand (FasL) and TRAIL [99]. These genes accelerate apoptotic cell death, especially in tubular epithelial cells, worsening AKI progression. Therefore, this unconventional NF-κB signaling pathway plays an essential role in regulating inflammation and apoptosis in AKI, and its dysregulation contributes to an imbalance between survival and cell death mechanisms. To support the idea that stress-dependent NF-κB signaling contributes to tubular injury, inflammation, and apoptosis in kidney injury models. A recent study reported that NINJ1-mediated plasma membrane rupture in renal tubular epithelial cells promotes damage-associated molecular pattern (DAMP) release, triggering inflammatory responses in AKI, suggesting amplification of NF-κB–linked inflammation after stress-induced tubular cell death [100]. Another study indicated that Josephin Domain Containing 2 (JOSD2) is protective against AKI by deubiquitinating SIRT7 and negatively controlling the SIRT7/NF-κB p65 inflammatory pathway in renal tubular epithelial cells [101]. Likewise, tetramethylpyrazine ameliorated sodium arsenite-induced AKI by restoring autophagic flux and reducing inflammatory injury, partly via YAP1-related stress signaling [102]. Another study reported the role of mitochondrial pyruvate carrier 2 that protects against cisplatin-induced AKI by enhancing mitochondrial metabolism and ameliorating renal tubular injury [103,104]. Collectively, the aforementioned research suggests that stress-dependent NF-κB signaling interplays with mitochondrial dysfunction, autophagy dysfunction, inflammatory cytokine generation, and tubular epithelial cell death in AKI.

### 3.6. Autophagy

Autophagy is an essential cellular process by which cells eliminate waste proteins and damaged organelles from the cells. Yoshinori Ohsumi was awarded the 2016 Nobel Prize in Physiology or Medicine for his seminal research on the basic mechanism of autophagy [105,106]. To date, the mechanism of autophagy and its interactions with various critical cellular processes have been documented in many studies, revealing its potential role in human disease [62,107,108]. Therefore, there is great interest in studying autophagy in the pathophysiology of various diseases, including AKI. The impact of NF-κB on autophagy dynamics in the setting of AKI is complex, affecting both the initiation and resolution stages of renal injury [63,108,109]. Emerging studies on AKI have demonstrated the activation of autophagy in animal models of AKI, such as ischemia–reperfusion (IR), cisplatin, and sepsis [6,110].

Most importantly, different stages of autophagy may have varying effects throughout the course of AKI. Early induction of autophagy and preservation of autophagic flux are likely to promote mitochondrial quality control and tubular cell survival. In contrast, defective autophagic flux, weakened mitophagy, or prolonged maladaptive autophagy may result in persistent inflammation, mitochondrial dysfunction, and tubular injury. NF-κB-mediated regulation of autophagy-related genes, such as Beclin-1, LC3 and Atg proteins, is also involved in the assessment of renal tubular cell fate following ischemia and nephrotoxic damage. Studies employing genetic and pharmacological techniques have revealed the potential role of autophagy in AKI [7,111]. In this regard, proximal tubule-specific (Atg5- or Atg7) conditional KO mice that received IR and cisplatin-induced acute kidney injury (AKI) consistently showed worse outcomes compared to wild-type mice, suggesting an indication for the pro-survival role of autophagy in AKI models [111,112,113]. In response to IR, proximal tubule-specific autophagy-deficient mice exhibited enhanced tubular damage, tubular cell apoptosis, mitochondrial damage, loss of renal function, and accumulation of p62 and ubiquitin-positive inclusion bodies [63,114,115]. Most of these autophagy processes are somehow linked with NF-κB signaling. Recent studies have indicated that NF-κB plays a key role in regulating genes involved in autophagy, such as Lcn2, Beclin-1, LC3 (microtubule-associated protein 1A/1B-light chain 3), and p62/SQSTM1 (sequestosome 1) [7,116,117]. In renal proximal tubular cells, recombinant lipocalin-2 (Lcn2) inhibits hypoxia-induced apoptosis by activation of the p65 subunit of the NF-κB. In general, autophagy acts as a safeguard against apoptosis; however, excessive autophagy induced by HIF-1α may lead to cell death. Lowering HIF-1α, preventing dysregulated autophagy activation, and suppressing apoptosis mediated by Lcn2 [118]. Hence, it is evident that NF-κB signaling-governed autophagy serves a protective function in AKI by alleviating renal damage through processes such as mitochondrial preservation and clearance of inclusion bodies, with genetic and pharmacological studies confirming its critical role in survival and repair mechanisms. Table 2 summarizes the cell-specific and temporal consequences of NF-κB activation in AKI.

## 4. Crosstalk Between Inflammation, Apoptosis, and Autophagy

Adequate modulation of NF-κB signaling is essential for preserving kidney homeostasis, since NF-κB is a central molecule that links inflammation, autophagy, and apoptosis in AKI. NF-κB functions as a context-dependent regulator where cellular stress may trigger apoptosis as well as autophagy in AKI. During the initial phases of AKI, autophagy has emerged as an essential defense mechanism, particularly in proximal tubule epithelial cells (PTECs), which activate autophagy-related genes. However, under extreme or prolonged stress, this process can be transformed into a cell death mechanism through apoptosis [6,121,122]. Previous mouse studies have reported that selective deletion of autophagy-related genes, such as *ATG5* or *ATG7*, results in significant kidney damage, which is attributed to an aggregation of damaged mitochondria and protein aggregates and tubulointerstitial fibrosis [119,123,124]. The accumulation of defective mitochondria and toxic aggregates, in turn, can activate tubular cell apoptosis, leading to cell death and enhanced renal injury.

**Figure 3:**
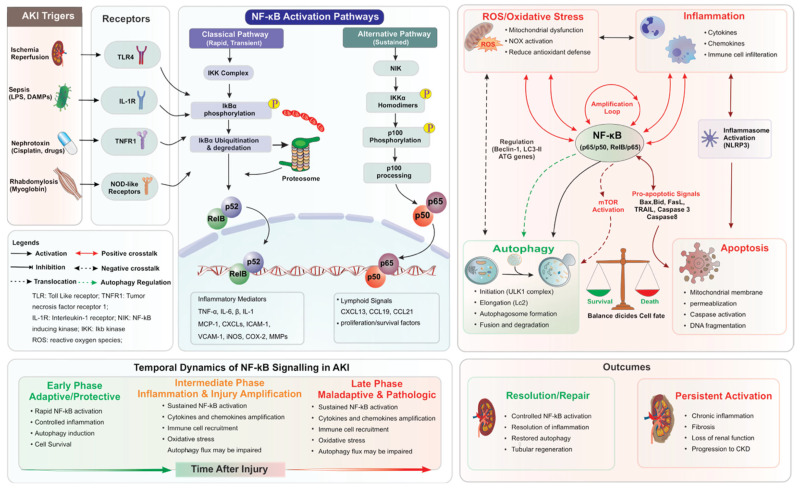
Stress-dependent integrative NF-κB signaling network in acute kidney injury. Upon stimulation of AKI-associated mediators, such as ischemia-reperfusion injury, sepsis, nephrotoxins, and rhabdomyolysis, cellular receptors are activated, leading to activation of canonical and non-canonical NF-κB signaling pathways. NF-κB activation modulates various chemokines, inflammasome, oxidative stress, autophagy, and apoptotic signaling via extensive bilateral crosstalk. Early transient activation of NF-κB results in adaptive inflammatory responses, such as autophagy and tissue repair, whereas sustained activation of NF-κB leads to persistent inflammation, oxidative stress, apoptosis, and fibrosis, leading to progression from AKI to chronic kidney disease (CKD).

Although renal cells typically exhibit a modest level of autophagy under normal physiological conditions, this mechanism becomes crucial when the kidneys are stressed or damaged. However, if the autophagic mechanism is compromised, NF-κB activation can stimulate apoptosis by increasing the expression of apoptotic genes such as Bax, Bid, Caspase-3, and Caspase-8 [125,126,127]. In contrast, autophagy may reduce NF-κB activity, thereby preventing apoptosis (Figure 3). The intricate connection between autophagy and apoptosis underscores the need to maintain a harmonious equilibrium, in which NF-κB acts as a crucial mediator that affects both pathways in the early stages of damage. These studies demonstrated that autophagy plays a vital role in maintaining cellular integrity under normal conditions and is also crucial for reducing damage and facilitating recovery during AKI. This association suggests that NF-κB can act as a bridge between autophagy and apoptosis in AKI. While it initially facilitates autophagic mechanisms to address cellular injury, its sustained activation can induce apoptosis, ultimately leading to cell death.

The NF-κB pathway plays a significant role in mediating autophagy and inflammation in AKI. In AKI, autophagy controls the PAMP- and DAMP-associated inflammatory response cascade. PAMPs stimulate toll-like receptors (TLRs) and trigger signaling pathways comprising mTOR. The complex interaction between NF-κB and mTOR regulates autophagy and inflammation in AKI [128,129]. Activation of NF-κB can hinder mTOR signaling, thereby facilitating autophagy initiation. Conversely, NF-κB-induced increase in pro-inflammatory cytokines, such as TNF-α, can trigger mTOR signaling and restrict autophagy [125,130]. Conversely, autophagy modulates the NF-κB pathway, which is crucial for the inflammatory response. Research indicates that limited autophagy aggravates inflammatory responses. In macrophages, the accumulation of ubiquitinated IkB, a significant inhibitor of NF-κB, is evident due to the blockage of the NF-κB pathway. Inflammatory cytokines IL-1β and IL-18 are produced in more substantial amounts under autophagy-inhibiting circumstances, such as the detection of Atg16L1, indicating autophagy in regulating NF-κB activation and consequent inflammation [131,132]. Furthermore, in sterile inflammation, such as ischemia or toxin exposure in AKI, autophagy is crucial in eliminating DAMPs produced by necrotic cells. It prevents excessive NF-κB-mediated inflammation. Also, the metabolic degradation of DAMPs, including dysfunctional mitochondria that produce ROS, is essential, as these ROS can trigger NF-κB activation, thereby perpetuating inflammation. Moreover, autophagy proteins like LC3B and Beclin-1 play a vital role in preserving mitochondrial integrity and restricting the translocation of mitochondrial DNA into the cytosol, hence avoiding inflammasome activation and subsequent amplification of NF-κB signaling and inflammation [133].

## 5. Future Research Directions

The current understanding of NF-κB’s role in the development and progression of AKI, by mediating inflammation, autophagy, and apoptosis and contributing to renal damage, makes it a potential therapeutic target. Targeting NF-κB signaling offers potential therapeutic avenues in AKI. More recently, translational and preclinical investigations have indicated that therapeutic regulation of NF-κB signaling can mitigate inflammation, oxidative stress, mitochondrial dysfunction and tubular apoptosis during AKI [15,45,93,134,135]. Several pharmaceutical treatments targeting TLR4/NF-κB signaling, inflammasome activation, ROS production and autophagy dysregulation have demonstrated renoprotective benefits in experimental models of AKI. These data indicate NF-κB signaling as a promising therapeutic target and suggest future exploration of NF-κB directed therapeutics to prevent AKI progression and improve renal recovery. To explore the role of NF-κB signaling in AKI, future research should encompass individual checkpoints in the NF-κB pathway. In this regard, various strategies have been developed to explore NF-κB’s role in human diseases. Recent studies have used IKK inhibitors and monoclonal antibodies targeting essential mediators of inflammation, such as IL-1, PD-1/PD-L1, and β2-microglobulin, to study NF-κB signaling [136,137,138,139]. Exploring the effectiveness of these inhibitors to improve NF-κB inhibition in AKI is essential. NF-κB activation is prevented by proteasome inhibitors such as carfilzomib, bortezomib, marizomib, and ixazomib [140,141,142,143]. It is vital to investigate the dual effects of next-generation proteasome inhibitors, such as marizomib, on NF-κB signaling in preclinical models of AKI to assess their impact on NF-κB signaling and cellular proteostasis. Also, there are methods to sequester NF-κB in the cytoplasm, such as blocking its nuclear translocation, using drugs such as tacrolimus, or using designed proteins like the IκBα super-repressor [144]. Investigate the utilization of IκBα super-repressor in preclinical models of AKI, with particular emphasis on NF-κB mediated renal inflammation and fibrosis. To minimize immunosuppressive side effects, it is imperative to test tacrolimus analogs that provide enhanced specificity for NF-κB inhibition. Moreover, DNA binding inhibitors like PPAR agonists and glucocorticoids that regulate the transcriptional activity of NF-κB have also been tested in another disease [145]. Therefore, examining the ability of PPARγ and PPARδ agonists to inhibit NF-κB-dependent pro-inflammatory and pro-fibrotic pathways in models of AKI should be meaningful. The use of tyrosine kinase inhibitors, which are upstream regulators of NF-κB, has been described in studies as a means of controlling NF-κB signaling [146]. For this reason, exploring the potential of new tyrosine kinase inhibitors in AKI, specifically focusing on their impact on inflammation and oxidative stress, could aid therapeutic development. Likewise, additional combination techniques involving anti-fibrotic drugs and tyrosine kinase inhibitors could be tested in models of AKI to CKD progression [53,147]. Also, the use of non-coding RNAs, including microRNAs and long non-coding RNAs, has recently been explored in NF-κB research and offers promising avenues for regulating NF-κB activity in AKI. Targeting NF-κB may reduce excessive inflammation that exacerbates renal damage, thereby improving patients’ clinical outcomes. Recent findings on the role of NF-κB in Klotho-Angiotensin (1-7)-NF-κB axis are critical, as Klotho is a protective protein and is downregulated in AKI [148]. Future treatment strategies designed to promote autophagy could be a promising option for addressing autophagy deficiencies linked to dysregulated inflammation during AKI. The application of autophagy stimulants, such as rapamycin or its equivalents, and autophagy inhibitors, such as 3-MA or chloroquine, can be explored as potential therapeutic agents for AKI.

LC3B and Beclin-1 are essential components of the autophagy pathway that play an important role in regulating the integrity of mitochondria and restricting the release of its DNA into the cytosol, which would trigger NF-κB-mediated inflammatory responses. Recent studies have reported the importance of autophagy-related genes such as LC3B and Beclin-1 in AKI [135,149,150]. These studies suggest that increasing autophagic activity might offer renal protection by reducing inflammation and cellular damage. In the context of AKI, the precise roles of LC3B and Beclin-1 in regulating NF-κB signaling remain poorly defined. Investigating how the regulation of mitochondrial health by LC3B and Beclin-1, and their interaction with NF-κB, could uncover novel therapeutic targets to reduce inflammation and tissue damage in AKI.

## 6. Conclusions

It is now accepted that NF-κB signaling bridges critical cellular processes, including inflammation, autophagy, and apoptosis, in the context of AKI. Through its role in regulating immune responses, NF-κB promotes innate and adaptive immune response pathways during AKI, increasing the expression of various inflammatory mediators (cytokines, chemokines, and inflammatory mediators) that exacerbate renal inflammation. This activation is mainly triggered by PRRs, such as DAMPs and TLRs, which activate NF-κB signaling, modulate the immune response, and enhance renal injury. It functions as an inducer of injury by regulating the expression of inflammatory cytokines and autophagic flux and by modulating apoptotic signaling. It represents a potential therapeutic target for future research. Moreover, to understand the cell-specific role of NF-κB and its temporal and spatial dynamics in AKI, modern techniques such as spatial transcriptomics and single-cell RNA sequencing will open new avenues. NF-κB plays a central role in AKI pathophysiology by initially governing autophagy-related gene expression, promoting cell survival, and contributing to cell death under prolonged stress. Future research should focus on integrating nanomaterial-based drug-delivery systems to address and mitigate the physiological challenges of targeting NF-κB in AKI-like complex conditions. Additionally, the complex and context-dependent nature of NF-κB signaling necessitates further research to refine therapeutic strategies that balance its pro-inflammatory, apoptotic, and autophagic roles. In this regard, a more comprehensive understanding of NF-κB’s interactions with other signaling pathways, including TGF-β and mTOR signaling, could identify synergistic approaches to modulate inflammation, increase autophagy, and decrease apoptosis in AKI. Thus, targeting NF-κB pathways offers promising therapeutic potential for modulating inflammation, reducing tissue damage, and promoting renal recovery in AKI.

## Figures and Tables

**Table 1:** Model-specific activation and biological consequences of NF-κB signaling pathways in acute kidney injury.

AKI Model	Major Trigger	Dominant NF-κB Axis	Major Cell Types	Key Mechanisms	References
Ischemia–reperfusion injury (IRI)	Hypoxia, ROS	Canonical NF-κB	Macrophages, tubular epithelial cells	Oxidative stress, inflammation, mitochondrial dysfunction	[48,49,57,58]
Sepsis-associated AKI	Systemic inflammation, LPS	TLR4/MyD88/NF-κB	Neutrophils, macrophages, endothelial cells	Cytokine storm, inflammasome activation, endothelial dysfunction	[39,40,59]
Cisplatin/nephrotoxic AKI	ROS, DNA damage	ROS–NF-κB pathway	Tubular epithelial cells	Mitochondrial dysfunction, apoptosis, defective autophagy	[6,60,61,62,63]
Rhabdomyolysis	Myoglobin, oxidative stress	TLR4/NF-κB	Tubular epithelial cells	ROS, inflammation, NOX activation	[64,65]

Representative references supporting the summarized mechanisms are shown.

**Table 2:** Cell-specific and temporal effects of NF-κB activation during acute kidney injury.

Cell Type	Early/Adaptive NF-κB Activation	Sustained/Maladaptive NF-κB Activation	Functional Consequence	References
Tubular epithelial cells	Stress adaptation, autophagy induction, survival signaling	Cytokine release, apoptosis, fibrosis-related signaling	Tubular injury and maladaptive repair	[6,112,113,119]
Macrophages	Debris clearance, innate immune activation	Persistent inflammation, ROS generation	Amplification of renal inflammation	[46,48,51,52]
Neutrophils	Initial antimicrobial response	Oxidative burst, protease release	Tissue injury and endothelial damage	[39,46,55]
Dendritic cells	Antigen presentation	Sustained immune activation	Adaptive immune amplification	[46,54]
T cells/Th17 cells	Cytokine regulation	Chronic inflammatory signaling	Persistent immune-mediated injury	[35,67,68]
Regulatory T cells (Tregs)	Immune suppression and resolution	Functional dysregulation	Impaired inflammation resolution	[35,46]
Endothelial cells	Vascular adaptation	Endothelial dysfunction, leukocyte adhesion	Microvascular injury and hypoxia	[39,69,120]

Representative references supporting the summarized mechanisms are shown.

## Data Availability

No new data were created or analyzed in this study.
